# Herpes simplex virus type 1 epidemiology in Latin America and the Caribbean: Systematic review and meta-analytics

**DOI:** 10.1371/journal.pone.0215487

**Published:** 2019-04-22

**Authors:** Layan Sukik, Maryam Alyafei, Manale Harfouche, Laith J. Abu-Raddad

**Affiliations:** 1 Infectious Disease Epidemiology Group, Weill Cornell Medicine-Qatar, Cornell University, Qatar Foundation—Education City, Doha, Qatar; 2 Department of Health Sciences, Qatar University, Doha, Qatar; 3 Department of Healthcare Policy and Research, Weill Cornell Medicine, Cornell University, New York, New York, United States of America; 4 College of Health and Life Sciences, Hamad bin Khalifa University, Doha, Qatar; University of Pretoria, SOUTH AFRICA

## Abstract

**Objectives:**

To investigate the epidemiology of herpes simplex virus type 1 (HSV-1) in Latin America and the Caribbean.

**Methods:**

Systematic review and meta-analytics guided by the Cochrane Collaboration Handbook and reported following the PRISMA guidelines.

**Results:**

Thirty-three relevant reports were identified including 35 overall (and 95 stratified) seroprevalence measures, and five and nine proportions of virus isolation in genital ulcer disease (GUD) and in genital herpes, respectively. Pooled mean seroprevalence was 57.2% (95% CI: 49.7–64.6%) among children and 88.4% (95% CI: 85.2–91.2%) among adults. Pooled mean seroprevalence was lowest at 49.7% (95% CI: 42.8–56.6%) in those aged ≤10, followed by 77.8% (95% CI: 67.9–84.8%) in those aged 10–20, 82.8% (95% CI: 73.1–90.8%) in those aged 20–30, 92.5% (95% CI: 89.4–95.1%) in those aged 30–40, and 94.2% (95% CI: 92.7–95.5%) in those aged ≥40. Age was the strongest source of heterogeneity in seroprevalence, explaining 54% of variation. Evidence was found for seroprevalence decline over time. Pooled mean proportion of HSV-1 isolation was 0.9% (95% CI: 0.0–3.6%) in GUD and 10.9% (95% CI: 4.4–19.4%) in genital herpes.

**Conclusions:**

HSV-1 is a widely prevalent infection in this region, but its epidemiology may be slowly transitioning, with still limited contribution for HSV-1 in genital herpes.

## Introduction

Infection with herpes simplex virus type 1 (HSV-1) is prevalent globally [1]. HSV-1 is responsible for a range of mild to serious morbidities [2, 3], with its typical clinical manifestation being orolabial herpes lesions [2, 4]. The infection, lifelong and mostly asymptomatic, is usually acquired orally and in childhood [3]. However, mounting evidence suggests an HSV-1 epidemiological transition in Europe and North America [4–7] and in Asia [8], associated with decreasing oral acquisition in childhood and increasing sexual acquisition (through oral sex) in adulthood [4–6]. In multiple Western countries, HSV-1 is already the primary cause of first episode genital herpes, surpassing the role of that of HSV-2 [4, 5, 7, 9–11]. An epidemiological transition is defined here as a significant change in the occurrence of the infection and/or its mode of transmission patterns.

HSV-1 infection is of growing interest and a focus of an international multi-sectorial effort, guided by the World Health Organization, to develop a vaccine to control infection transmission [12, 13]. To inform these global health efforts, we aimed in the present study to provide a detailed investigation of the epidemiology of HSV-1 in Latin America and the Caribbean, by conducting a comprehensive systematic review and a range of meta-analytics. Importantly, we estimated HSV-1 antibody prevalence (seroprevalence), its associations and temporal trend, and assessed the role of HSV-1 as a cause of clinically-diagnosed genital ulcer disease (GUD) and clinically-diagnosed genital herpes.

## Material and methods

The methodology of this study was adapted from that of a study investigating HSV-1 epidemiology in Asia [8].

### Data sources and search strategy

The systematic review and meta-analyses were guided by the Cochrane collaboration Handbook [14], and were reported following the Preferred Reporting Items for Systematic Reviews and Meta-analyses (PRISMA) guidelines (checklist in S1 Table) [15]. PubMed, Embase, and LILACS databases were systematically searched up to September 12, 2018. The search strategies included MeSH/Emtree and broad terms with no language or year restrictions (S2 Table). The definition for the Latin America and the Caribbean region included 46 countries, as listed in S1 Box.

### Study selection and inclusion and exclusion criteria

Search results were de-duplicated using a reference manager, Endnote (Thomson Reuters, USA). Titles and abstracts were screened for relevant and potentially relevant reports, and the full-texts of these relevant or potentially relevant reports were retrieved for further screening. Bibliographies of identified relevant reports and reviews were also screened for additional potentially relevant reports. Initial screening was conducted by LS and MA, and double screening was conducted by MH.

Reports met the inclusion criteria if they reported primary data on any of three outcome measures: 1) HSV-1 seroprevalence based on a valid diagnostic method (i.e. strictly type-specific glycoprotein-G based assays), 2) proportion of HSV-1 virus isolation in clinically-diagnosed GUD, or 3) proportion of HSV-1 virus isolation in clinically-diagnosed genital herpes.

Only measures with a sample size ≥10 were included. Case reports, editorials, letters to editors, commentaries, and reviews were excluded. HSV-1 seroprevalence measures among newborns <3 months of age were excluded, as they may reflect maternal antibodies as opposed to current infection.

In this systematic review, a “report” denotes a publication reporting a relevant outcome measure, while a “study” denotes the extracted details of an outcome measure.

### Data extraction and synthesis

Relevant reports were extracted by LS and MA, and double-extracted by MH. Extracted data included publication details, population characteristics, study methodology characteristics, and outcome measures. The extracted variables are listed in S2 Box. Extracted overall outcome measures for the full sample were replaced by stratified measures (if available), based on a pre-defined protocol for the stratification hierarchy, provided that the sample size in each stratum was ≥10.

**Fig 1:**
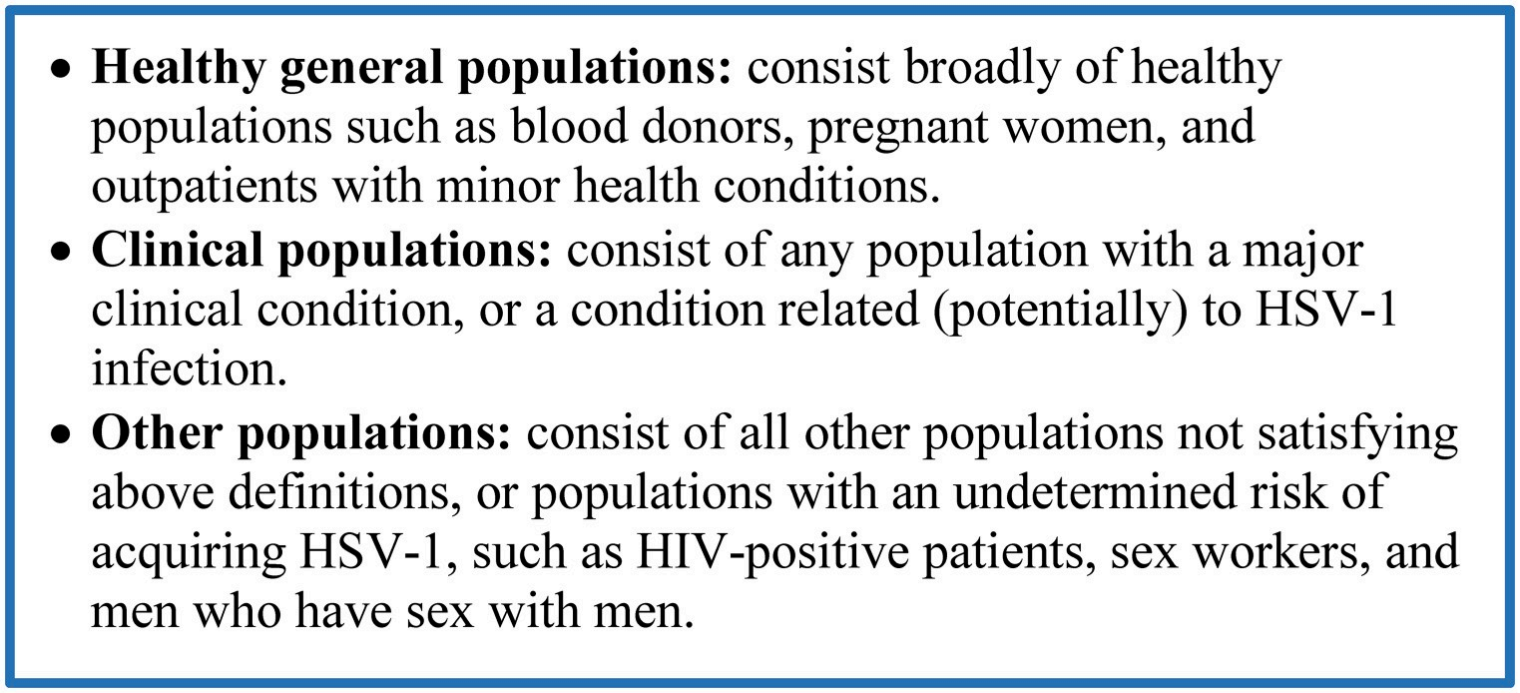
Population type definition and classification. Abbreviation: HSV-1 = Herpes simplex virus type 1.

For HSV-1 seroprevalence measures, extracted strata were prioritized for population type (Fig 1), followed by age bracket (children (≤15 years of age) *versus* adults (>15 years of age)), and age group (≤10, 10–20, 20–30, 30–40, and ≥40 years of age). These age ranges were informed by the actually available age strata in extracted studies. For the proportions of HSV-1 virus isolation in GUD or in genital herpes, the stratification hierarchy included primary *versus* recurrent episode, followed by study site (hospital *versus* sexually transmitted infection clinic).

### Quality assessment

Given the documented limitations in the sensitivity and specificity of HSV-1 serology diagnostic assays [16, 17], the validity of the type-specific diagnostic method of each study was investigated and determined in consultation with an expert advisor in HSV-1 serology, Professor Rhoda Ashley-Morrow, University of Washington, Seattle. Studies where the validity of the diagnostic method could not be confirmed, were excluded from the systematic review and meta-analytics.

Informed by the Cochrane approach [14], studies with valid assays were further classified into low *versus* high precision based on the number of individuals tested for HSV-1 in that study (<100 *versus* ≥100). Moreover, studies were classified into low *versus* high risk of bias (ROB) using two quality domains: sampling method (probability-based *versus* non-probability-based sampling) and response rate (≥80% *versus* <80%). Studies with no information on a quality domain were classified as having an “unclear” ROB for that domain.

Precision and ROB domains were included in the meta-regression analyses (as described below), to examine their associations with seroprevalence, that is the influence of the characteristics of the study methodology on observed HSV-1 seroprevalence.

### Meta-analyses

Pooled means were estimated for HSV-1 seroprevalence and its relevant strata by population type, age bracket, age group, and year of publication category (<2000, 2000–2009, and 2010–2018), as well as for the proportions of HSV-1 virus isolation in GUD and in genital herpes, whenever ≥3 measures were available. The estimates were calculated in R version 3.4.1 [18] using a DerSimonian-Laird random-effects model [19], as applied in the meta package [20]. The Freeman-Tukey type arcsine square-root transformation [21] was utilized to stabilize the variance of each included measure. Forest plots were produced to visualise estimates and their 95% confidence intervals (CIs).

Heterogeneity was assessed using three complementary metrics: 1) Cochrane Q statistics to test for existence of heterogeneity [19, 22], 2) I^2^ to provide the magnitude of heterogeneity that is explained by true differences in the outcome measures across studies (as opposed to being due to sampling variation) [19, 23], and 3) prediction interval to provide the range of true effect sizes of the outcome measures around the pooled mean [19, 23].

### Meta-regressions

Associations with HSV-1 seroprevalence and sources of between-study heterogeneity were investigated using univariable and multivariable random-effects meta-regression analyses. Independent variables with a p-value ≤0.1 in univariable analysis were included in the multivariable analyses. In the multivariable models, a p-value of ≤0.05 for any given independent variable indicated strong evidence for an association with HSV-1 seroprevalence.

The included independent variables were set *a priori* and consisted of: age bracket, age group, sex, population type, country’s income, assay type (Western blot, enzyme-linked immunosorbent assay, and others), sample size (<100 *versus* ≥100), sampling method (non-probability-based *versus* probability-based), response rate (≥80 *versus* otherwise), year of publication category, year of data collection, and year of publication.

The variable of country’s income (for countries with available data and per World Bank classification [24]) categorized the countries into upper-middle-income countries (Brazil, Colombia, Costa Rica, Jamaica, Mexico, and Peru), high-income countries (Barbados, Chile, and Argentina), and “mixed” for studies including different countries in the study sample.

Missing values for the year of data collection were imputed utilizing data for the year of publication as adjusted by the median difference between year of publication and year of data collection (for studies with non-missing data).

The meta-regressions were conducted on the log-transformed proportions (with inverse-variance weighting) in Stata/SE version 13 [25], using the metareg package [26].

## Results

### Search results and scope of evidence

**Fig 2:**
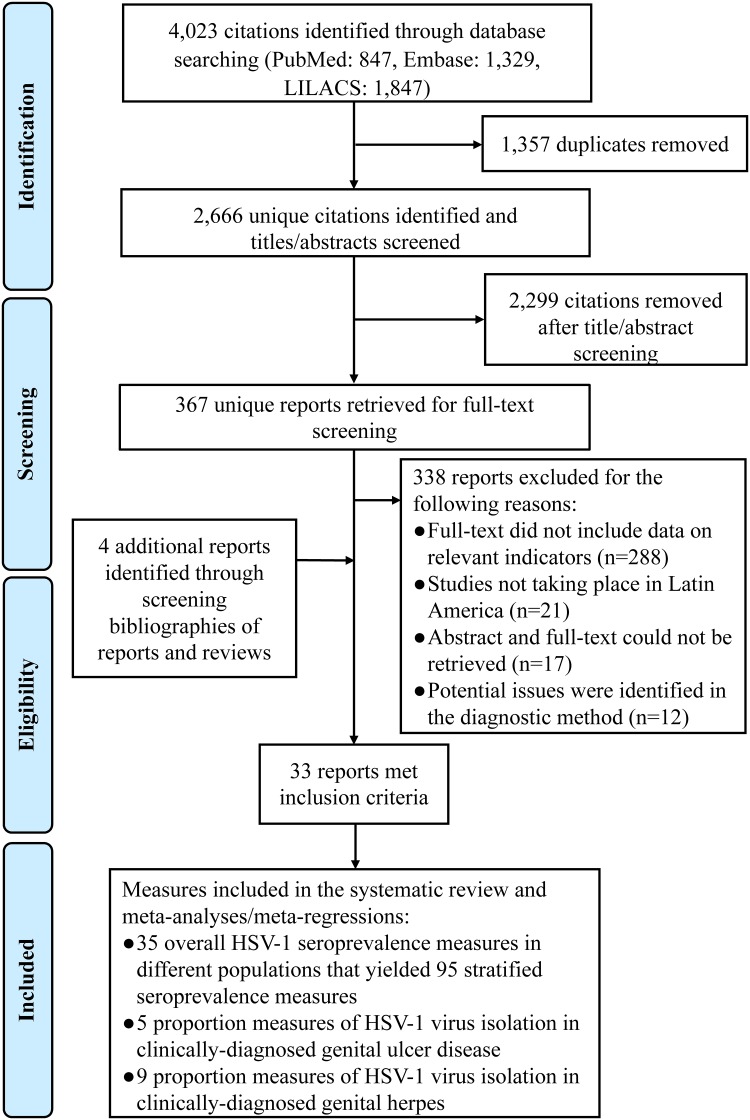
Flow chart of article selection for the systematic review of HSV-1 infection in Latin America and the Caribbean, per the PRISMA guidelines [15]. Abbreviation: HSV-1 = Herpes simplex virus type 1.

Fig 2 details the study selection process per PRISMA guidelines [15]. The search identified 4,023 citations (PubMed: 847, Embase: 1,329, and LILACS 1,847) of which duplicates were removed. Title and abstract screening yielded 367 relevant and potentially relevant reports. Full-text screening of these latter reports identified 29 reports that met the inclusion criteria. Four additional relevant reports [27–30] were identified through bibliography screening of reviews and relevant reports.

Extracted measures included: 35 overall HSV-1 seroprevalence measures yielding 95 stratified seroprevalence measures, five proportions of viral HSV-1 isolation in GUD, and nine proportions of viral HSV-1 isolation in genital herpes. No HSV-1 seroprevalence measure was identified among clinical children populations.

### Overview of HSV-1 seroprevalence

Table 1 lists the extracted stratified HSV-1 seroprevalence measures and their characteristics (number of measures (n) = 95). Most measures were from studies conducted prior to 2010 (n = 76; 80.0%), and were based on convenience samples (n = 68; 71.0%). Seroprevalence across all measures ranged between 7.7–100% with a median of 86.0% (n = 95; Table 2).

**Table 1:** Studies reporting HSV-1 seroprevalence in Latin America and the Caribbean.

Author, year	Year(s) of data collection	Country	Study site	Study design	Sampling method	Population	HSV-1 serological assay	Sample size	HSV-1 seroprevalence (%)
**Healthy children populations (n = 19)**									
Clemens, 2010 [43]	1996–97	Brazil	Community	CS	RS	1–5 years old boys	ELISA	52	44.2
Clemens, 2010 [43]	1996–97	Brazil	Community	CS	RS	6–10 years old boys	ELISA	49	55.1
Clemens, 2010 [43]	1996–97	Brazil	Community	CS	RS	11–15 years old boys	ELISA	125	65.6
Clemens, 2010 [43]	1996–97	Brazil	Community	CS	RS	1–5 years old girls	ELISA	47	38.3
Clemens, 2010 [43]	1996–97	Brazil	Community	CS	RS	6–10 years old girls	ELISA	50	58.0
Clemens, 2010 [43]	1996–97	Brazil	Community	CS	RS	11–15 years old girls	ELISA	126	74.6
Conde-Glez, 2013 [44]	2005–06	Mexico	Community	CS	RS	1–9 years old girls	ELISA	252	51.0
Conde-Glez, 2013 [44]	2005–06	Mexico	Community	CS	RS	1–9 years old boys	ELISA	264	50.0
Cowan, 2003 [45]	-	Brazil	Outpatient clinic	CS	Conv	1–4 years old children	ELISA	232^a^	36.0
Cowan, 2003 [45]	-	Brazil	Outpatient clinic	CS	Conv	5–9 years old children	ELISA	232^a^	52.4
Cowan, 2003 [45]	-	Brazil	Outpatient clinic	CS	Conv	10–14 years old children	ELISA	233^a^	68.1
De Salles-Gomes, 1981 [46]	1980	Brazil	Outpatient clinic	CS	Conv	7–11 month babies	IF	13	7.7
De Salles-Gomes, 1981 [46]	1980	Brazil	Outpatient clinic	CS	Conv	1–4 years old children	IF	50	38.0
De Salles-Gomes, 1981 [46]	1980	Brazil	Outpatient clinic	CS	Conv	5–9 years old children	IF	50	64.0
De Salles-Gomes, 1981 [46]	1980	Brazil	Outpatient clinic	CS	Conv	10–14 years old children	IF	50	92.0
Robinson, 2002 [47]	-	Multiple countries in South America	Community	CS	Conv	≤3 years old children	WB	23	29.0
Robinson, 2002 [47]	-	Multiple countries in South America	Community	CS	Conv	4–6 years old children	WB	56	72.0
Robinson, 2002 [47]	-	Multiple countries in South America	Community	CS	Conv	7–9 years old children	WB	68	76.0
Robinson, 2002 [47]	-	Multiple countries in South America	Community	CS	Conv	10–13 years old children	WB	54	81.0
**Healthy adult populations (n = 51)**									
Arriaga-Demeza, 2008 [48]	2002–03	Mexico	Community	CS	Conv	18–20 years old females	WB	195	50.3
Arriaga-Demeza, 2008 [48]	2002–03	Mexico	Community	CS	Conv	21–25 years old females	WB	153	53.6
Arriaga-Demeza, 2008 [48]	2002–03	Mexico	Community	CS	Conv	≥26 years old females	WB	31	74.2
Arriaga-Demeza, 2008 [48]	2002–03	Mexico	Community	CS	Conv	18–20 years old males	WB	103	46.6
Arriaga-Demeza, 2008 [48]	2002–03	Mexico	Community	CS	Conv	21–25 years old males	WB	102	61.8
Arriaga-Demeza, 2008 [48]	2002–03	Mexico	Community	CS	Conv	≥26 years old males	WB	18	55.6
Morrow, 2014 [16]	2000–01	Argentina	Community	CS	Conv	Argentinian women	WB	99	98.9
Morrow, 2014 [16]	2000–01	Costa Rica	Community	CS	Conv	Costa Rican women	WB	98	92.9
Morrow, 2014 [16]	2000–01	Mexico	Community	CS	Conv	Mexican women	WB	100	98.0
Clemens, 2010 [43]	1996–97	Brazil	Community	CS	RS	16–20 years old males	ELISA	119	69.8
Clemens, 2010 [43]	1996–97	Brazil	Community	CS	RS	21–30 years old males	ELISA	107	76.6
Clemens, 2010 [43]	1996–97	Brazil	Community	CS	RS	31–40 years old males	ELISA	78	85.9
Clemens, 2010 [43]	1996–97	Brazil	Community	CS	RS	16–20 years old females	ELISA	128	75.8
Clemens, 2010 [43]	1996–97	Brazil	Community	CS	RS	21–30 years old females	ELISA	126	81.0
Clemens, 2010 [43]	1996–97	Brazil	Community	CS	RS	31–40 years old females	ELISA	82	81.7
Conde-Glez, 2013 [44]	2005–06	Mexico	Community	CS	RS	20–29 years old females	ELISA	252^a^	78.0
Conde-Glez, 2013 [44]	2005–06	Mexico	Community	CS	RS	30–39 years old females	ELISA	252^a^	96.0
Conde-Glez, 2013 [44]	2005–06	Mexico	Community	CS	RS	40–49 years old female	ELISA	252^a^	91.0
Conde-Glez, 2013 [44]	2005–06	Mexico	Community	CS	RS	50–59 years old females	ELISA	252^a^	98.0
Conde-Glez, 2013 [44]	2005–06	Mexico	Community	CS	RS	≥60 years old females	ELISA	252^a^	95.0
Conde-Glez, 2013 [44]	2005–06	Mexico	Community	CS	RS	20–29 years old males	ELISA	264^a^	91.0
Conde-Glez, 2013 [44]	2005–06	Mexico	Community	CS	RS	30–39 years old males	ELISA	264^a^	91.0
Conde-Glez, 2013 [44]	2005–06	Mexico	Community	CS	RS	40–49 years old male	ELISA	264^a^	93.0
Conde-Glez, 2013 [44]	2005–06	Mexico	Community	CS	RS	50–59 years old males	ELISA	264^a^	95.0
Conde-Glez, 2013 [44]	2005–06	Mexico	Community	CS	RS	≥60 years old males	ELISA	264^a^	94.0
Corona, 2010 [28]	2002–05	Mexico	Community	CS	Conv	≥ 26 years old students	ELISA	59	72.9
Corona, 2010 [28]	2002–05	Mexico	Community	CS	Conv	21–25 years old students	ELISA	412	59.7
Corona, 2010 [28]	2002–05	Mexico	Community	CS	Conv	18–20 years old students	ELISA	335	50.1
Cowan, 2003 [45]	-	Brazil	Outpatient clinic	CS	Conv	15–19 years old adults	ELISA	146	83.3
Cowan, 2003 [45]	-	Brazil	Outpatient clinic	CS	Conv	20–29 years old adults	ELISA	147^a^	83.6
Cowan, 2003 [45]	-	Brazil	Outpatient clinic	CS	Conv	30–34 years old adults	ELISA	147^a^	95.2
Cowan, 2003 [45]	-	Brazil	Outpatient clinic	CS	Conv	35–39 years old adults	ELISA	147^a^	92.9
Cowan, 2003 [45]	-	Brazil	Outpatient clinic	CS	Conv	40–44 years old adults	ELISA	147^a^	96.0
Cowan, 2003 [45]	-	Brazil	Outpatient clinic	CS	Conv	≥45 years old adults	ELISA	147^a^	94.6
De Salles-Gomes, 1981 [46]	1980	Brazil	Outpatient clinic	CS	Conv	15–19 years old adults	IF	50	90.0
De Salles-Gomes, 1981 [46]	1980	Brazil	Outpatient clinic	CS	Conv	20–24 years old adults	IF	50	84.0
De Salles-Gomes, 1981 [46]	1980	Brazil	Outpatient clinic	CS	Conv	25–29 years old adults	IF	50	86.0
De Salles-Gomes, 1981 [46]	1980	Brazil	Outpatient clinic	CS	Conv	30–34 years old adults	IF	60	98.3
De Salles-Gomes, 1981 [46]	1980	Brazil	Outpatient clinic	CS	Conv	35–39 years old adults	IF	50	90.0
De Salles-Gomes, 1981 [46]	1980	Brazil	Outpatient clinic	CS	Conv	≥40 years old adults	IF	50	96.0
Evans, 1974 [49]	-	Brazil	Outpatient clinic	CC^C^	Conv	Healthy adults	IF	26	87.5
Jimemez, 1979 [50]	-	Costa Rica	Outpatient clinic	CS	Conv	≥18 years old students	NAb	16	50.0
Levett, 2005 [51]	-	Barbados	Outpatient clinic	CS	Conv	Blood donors	ELISA	184	81.0
Levett, 2005 [51]	-	Barbados	Outpatient clinic	CS	Conv	Ante-natal clinic attendees	ELISA	122	83.6
Lupi, 2011 [52]	1996–97	Brazil	Outpatient clinic	Cohort^b^	Conv	Blood donors	ELISA	155	68.0
Oberle, 1989 [53]	1984–85	Costa Rica	Community	CS	MCS	≥25 years old females	MAb	766	97.1
Patnaik, 2007 [54]	1985–97	Peru	Community	CS	Conv	Peruvian women	WB	171	91.8
Patnaik, 2007 [54]	1985–97	Colombia	Community	CS	Conv	Colombian women	WB	65	89.2
Prabhakar, 1984 [55]	-	Jamaica	Hospital	CC^C^	Conv	Healthy Jamaican women	NAb	60	38.3
Smith, 2002 [29]	1996–97	Peru	Hospital	CC^C^	Conv	Healthy Peruvian women	WB	171	91.8
Smith, 2002 [29]	1985–88	Colombia	Community	CC^C^	Conv	Healthy Colombian women	WB	65	89.2
**Healthy age-mixed populations (n = 2)**									
Conde-Glez, 2013 [44]	2005–06	Mexico	Community	CS	RS	10–19 years old females	ELISA	252	70.0
Conde-Glez, 2013 [44]	2005–06	Mexico	Community	CS	RS	10–19 years old males	ELISA	264	71.0
**Clinical adult populations (n = 7)**									
Calderon, 2018 [56]	2014–15	Peru	Outpatient clinic	CS	Conv	Women with breast cancer	ELISA	44	88.6
Evans, 1974 [49]	-	Brazil	Outpatient clinic	CC^C^	Conv	Patients with Hodgkin’s disease	IF	26	84.4
Moreira, 2018 [57]	2015–16	Brazil	Outpatient clinic	CC^C^	Conv	Women from a highly ZIKV-affected region	WB	32	93.8
Moreira, 2018 [57]	2015–16	Brazil	Outpatient clinic	CC^C^	Conv	Women from a highly ZIKV-affected region	WB	160	95.0
Smith, 2002 [29]	1996–97	Peru	Hospital	CC^C^	Conv	Women with squamous-cell carcinoma	WB	166	91.5
Smith, 2002 [29]	1996–97	Peru	Hospital	CC^C^	Conv	Women with adeno-squamous carcinoma	WB	24	100
Smith, 2002 [29]	1985–88	Colombia	Hospital	CC^C^	Conv	Women with squamous-cell carcinoma	WB	78	74.4
**Other populations (n = 16)**									
Levett, 2005 [51]	-	Barbados	Outpatient clinic	CS	Conv	HIV-positive adults	ELISA	120	89.2
Luchsinger, 2010 [58]	2005–06	Chile	Outpatient clinic	CS	Conv	HIV-positive adults	ELISA	400	92.2
Boulos, 1992 [59]	-	Haiti	Outpatient clinic	CS	Conv	Healthy/clinical women	ELISA	228	96.9
Conde-Glez, 1999 [60]	1992	Mexico	Outpatient clinic	CS	Conv	16–22 years old FSWs	WB	302	92.7
Conde-Glez, 1999 [60]	1992	Mexico	Outpatient clinic	CS	Conv	23–27 years old FSWs	WB	330	93.1
Conde-Glez, 1999 [60]	1992	Mexico	Outpatient clinic	CS	Conv	28–32 years old FSWs	WB	187	94.7
Conde-Glez, 1999 [60]	1992	Mexico	Outpatient clinic	CS	Conv	33–37 years old FSWs	WB	101	94.1
Conde-Glez, 1999 [60]	1992	Mexico	Outpatient clinic	CS	Conv	>37 years old FSWs	WB	77	100
Duenas, 1972 [61]	-	Colombia	Outpatient clinic	CS	Conv	14–15 years old FSWs	NAb	15	100
Duenas, 1972 [61]	-	Colombia	Outpatient clinic	CS	Conv	16–17 years old FSWs	NAb	56	100
Duenas, 1972 [61]	-	Colombia	Outpatient clinic	CS	Conv	18–19 years old FSWs	NAb	43	100
Duenas, 1972 [61]	-	Colombia	Outpatient clinic	CS	Conv	20–21 years old FSWs	NAb	34	100
Duenas, 1972 [61]	-	Colombia	Outpatient clinic	CS	Conv	22–25 years old FSWs	NAb	46	100
Duenas, 1972 [61]	-	Colombia	Outpatient clinic	CS	Conv	26–35 years old FSWs	NAb	95	100
Duenas, 1972 [61]	-	Colombia	Outpatient clinic	CS	Conv	≥36 years old FSWs	NAb	54	100
Lupi, 2011 [52]	1996–97	Brazil	Hospital	Cohort^b^	Conv	Men who have sex with men	ELISA	170	85.0

^a^ Study included sample size only for the total sample, but not for the strata. Each stratum sample size was set at total sample size divided by the number of strata.

^b^ The original study design of the study is prospective cohort. The included seroprevalence measures are those for the baseline measures at the onset of the study, before start of follow-up.

^c^ The original study design of the study is case-control. The included seroprevalence measures are those for each of cases and controls, separately. The population type classification was assigned based on the actual population type for each of cases and controls, separately.

Abbreviations: Conv = Convenience, CS = Cross-sectional, CC = Case-control, ELISA = Enzyme-linked immunosorbent type-specific assay, FSWs = Female sex workers, HIV = Human immunodeficiency virus, HSV-1 = Herpes simplex virus type 1, IF = Indirect immunofluorescence, MAb = Monoclonal antibody, MCS = Multistage cluster sampling, NAb = Neutralizing antibody, RS = Random sampling, WB = Western blot, ZIKV = Zika virus.

**Table 2:** Pooled mean estimates for HSV-1 seroprevalence in Latin America and the Caribbean.

Population type	Outcome measures	Samples	HSV-1 seroprevalence		Pooled mean HSV-1 seroprevalence	Heterogeneity measures		
	Total n	Total N	Range	Median	Mean (95% CI)	Q^a^ (p-value)	I^2^^b^ (%) (95% CI)	Prediction^c^ Interval (%)
**Healthy general populations**								
Children	19	2,026	7.7–92.0	55.1	57.2 (49.7–64.6)	190.8 (p<0.001)	90.6 (86.8–93.3)	24.7–86.7
Adults	51	7,917	38.3–98.9	87.6	84.5 (79.9–88.5)	1,323.5 (p<0.001)	96.2 (95.7–96.8)	46.1–100
Age-mixed	2	516	70.0–71.0	70.5	70.3 (66.2–74.2) ^d^	-	-	-
All healthy general populations	72	10,459	7.7–98.9	81.0	77.7 (72.9–82.2)	2,269.1 (p<0.001)	96.9 (96.5–97.3)	32.6–100
**Clinical populations**								
Adults	7	530	74.4–100	91.5	90.9 (84.2–95.9)	25.9 (p<0.001)	76.8 (51.5–88.9)	65.5–100
All clinical populations	7	530	74.4–100	91.5	90.9 (84.2–95.9)	25.9 (p<0.001)	76.8 (51.5–88.9	65.5–100
**Other populations**								
HIV positive patients	2	520	89.2–92.2	90.7	91.5 (88.8–93.7)^d^	-	-	-
Female sex workers	12	1,340	93.1–100	100	98.5 (96.4–99.8)	46.3 (p<0.001)	76.2 (58.4–96.4)	88.4–100
Men who have sex with men	1	170	-	-	85.3 (79.1–90.2)^d^	-	-	-
Mixed healthy/clinical adults populations	1	228	-	-	96.9 (93.8–98.7)^d^	-	-	-
**Age group**								
≤10 years	14	1,438	7.7–76.0	50.5	49.7 (42.8–56.6)	76.4 (p<0.001)	83.0 (72.7–89.4)	24.8–74.7
10–20 years	17	2,294	46.6–100	74.6	77.8 (67.9–84.8)	280.8 (p<0.001)	94.3 (92.2–95.8)	40.0–99.5
20–30 years	12	1,926	53.6–100	82.5	82.8 (73.1–90.8)	276.9 (p<0.001)	96.0 (94.5–97.2)	39.1–100
30–40 years	9	1,181	81.7–98.3	92.9	92.5 (89.4–95.1)	24.6 (p = 0.002)	67.4 (34.3–83.8)	81.4–99.0
≥40 years	11	2,128	89.2–98.0	94.6	94.2 (92.7–95.5)	17.5 (p = 0.064)	42.9 (0.0–71.8)	89.9–97.4
Mixed	32	4,280	38.3–100	90.3	89.6 (85.7–93.9)	405.7 (p<0.001)	92.4 (90.2–94.0)	62.9–100
**Age bracket**								
All children	19	2,026	7.7–92.0	55.1	57.2 (49.7–64.6)	190.8 (p<0.001)	90.6 (86.8–93.3)	24.7–86.7
All adults	73	10,690	38.3–100	91.0	88.4 (85.2–91.2)	1,588.2 (p<0.001)	95.5 (94.8–96.0)	54.8–100
All age-mixed	3	531	70.0–100	71.0	77.5 (65.8–87.5)	12.3 (p = 0.002)	83.7 (50.8–94.6)	0.0–100
**Year of publication category**								
<2000	28	2,935	7.7–100	93.6	90.8 (85.8–94.9)	394.7 (p<0.001)	93.2 (91.2–94.7)	57.1–100
2000–2009	32	3,844	29.0–100	83.0	80.7 (73.6–87.0)	847.7 (p<0.001)	96.3 (95.6–97.0)	34.0–100
2010–2018	35	6,468	38.3–98.0	78.0	78.8 (72.7–84.3)	1,113.4 (p<0.001)	96.9 (96.4–97.4)	37.4–100
**All studies**	**95**	**13,335**	**7.7–100**	**86.0**	**83.1 (79.3–86.5)**	**2,772.4 (p<0.001)**	**96.6 (96.2–97.0)**	**40.2–100**

^a^ Q: The Cochran’s Q statistic is a measure assessing the existence of heterogeneity in pooled outcome measures, here HSV-1 seroprevalence.

^b^ I^2^: A measure assessing the magnitude of between-study variation that is due to true differences in HSV-1 seroprevalence across studies rather than sampling variation.

^c^ Prediction interval: A measure quantifying the distribution 95% interval of true HSV-1 seroprevalence around the estimated pooled mean.

^d^ No meta-analysis was done as number of studies was <3. If there was only one study, the reported 95% CI is the 95% CI of this study. If there were two studies, both samples were merged to yield one sample size, for which the 95% CI was calculated.

Abbreviations: CI = Confidence interval, HIV = Human immunodeficiency virus, HSV-1 = Herpes simplex virus type 1.

HSV-1 seroprevalence ranged between 7.7–92.0% with a median of 55.1% among healthy children populations (n = 19), between 38.3–98.9% with a median of 87.6% among healthy adult populations (n = 51), and between 74.4–100% with a median of 91.5% among clinical adult populations (n = 7). Table 2 lists summaries for other population categories.

### Pooled mean estimates for HSV-1 seroprevalence

Table 2 displays the results of the meta-analyses. The overall pooled mean HSV-1 seroprevalence (n = 95) was 83.1% (95% CI: 79.2–86.5%).

The pooled mean HSV-1 seroprevalence was 57.2% (95% CI: 49.7–64.6%) among healthy children populations, 84.5% (95% CI: 79.9–88.5%) among healthy adult populations, and 90.9% (95% CI: 84.2–95.9%) among clinical adult populations.

The pooled mean seroprevalence increased with age. It was lowest at 49.7% (n = 14; 95% CI: 42.8–56.6%) in those aged ≤10, followed by 77.8% (n = 17; 95% CI: 67.9–84.8%) in those aged 10–20, 82.8% (n = 12; 95% CI: 73.1–90.8%) in those aged 20–30, 92.5% (n = 9; 95% CI: 89.4–95.1%) in those aged 30–40, and 94.2% (n = 11; 95% CI: 92.7–95.5%) in those aged ≥40.

The pooled mean seroprevalence decreased with time. It was highest at 90.8 (95% CI: 85.8–94.9%) before the year 2000, followed by 80.7% (95% CI: 73.6–87.0%) in 2000–2009, and 78.8% (95% CI: 72.7–84.3) in 2010–2018.

Forest plots for all adult populations and all children populations can be found in S1 Fig. All meta-analyses showed evidence of heterogeneity (Table 2). Heterogeneity was attributed to true variability in seroprevalence across studies rather than chance (Table 2). The heterogeneity was affirmed by the wide prediction intervals (Table 2).

### Predictors of HSV-1 seroprevalence

Table 3 and S3 Table display the results of the univariable and multivariable analyses. In the univariable analyses, age bracket, age group, sex, population type, year of publication category, year of data collection, and year of publication qualified to be included in the multivariable analysis (p<0.1). Country’s income, assay type, response rate, sample size, and sampling method all had a p-value >0.1, and hence, were not included in the multivariable analyses.

**Table 3:** Univariable and multivariable meta-regression models for HSV-1 seroprevalence in Latin America and the Caribbean.

			Outcome measures	Samples	Univariable analysis			Multivariable analysis^a^			
								Model 1^a^		Model 2^b^	
			Total n	Total N	*RR* (95%CI)	p-value	Adjusted R^2^ (%)	*ARR* (95%CI)	p-value	*ARR* (95%CI)	p-value
**Population Characteristics**	**Age bracket**	Children	19	2,026	1.00	-		1.00	-	-	-
		Adults	73	10,690	1.45 (1.29–1.64)	<0.001		1.39 (1.24–1.57)	<0.001	-	-
		Age-mixed	3	531	1.35 (1.04–1.75)	0.022	35.37	1.30 (1.00–1.67)	0.042	-	-
	**Age group**	≤10	14	1,438	1.00	-		-	-	1.00	-
		10–20	17	2,294	1.44 (1.24–1.67)	<0.001		-	-	1.36 (1.19–1.56)	<0.001
		20–30	12	1,926	1.53 (1.31–1.79)	<0.001		-	-	1.44 (1.25–1.65)	<0.001
		30–40	9	1,181	1.76 (1.49–2.08)	<0.001		-	-	1.70 (1.47–1.97)	<0.001
		≥40	11	2,128	1.81 (1.54–2.11)	<0.001		-	-	1.81 (1.58–2.08)	<0.001
		Mixed	32	4,280	1.68 (1.47–1.93)	<0.001	53.99	-	-	1.54 (1.35–1.75)	<0.001
	**Sex**	Female	46	6,723	1.00	-		1.00	-	1.00	-
		Male	17	2,771	0.86 (0.75–1.00)	0.053		0.96 (0.85–1.09)	0.572	0.97 (0.88–1.07)	0.557
		Mixed	32	3,751	0.93 (0.82–1.05)	0.277	3.62	1.03 (0.92–1.14)	0.618	1.00 (0.92–1.09)	0.956
	**Population type**	Healthy	72	10,456	1.00	-		1.00	-	1.00	-
		Clinical	7	530	1.19 (0.99–1.43)	0.062		1.10 (0.93–1.29)	0.249	1.12 (0.97–1.28)	0.116
		Other	16	2,258	1.28 (1.12–1.45)	<0.001	17.13	1.15 (1.01–1.31)	0.035	1.16 (1.04–1.29)	0.006
	**Country’s income**	UMIC	85	11,891	1.00	-		-	-	-	-
		HIC	5	925	1.12 (0.88–1.42)	0.324		-	-	-	-
		Other^c^	5	429	0.95 (0.73–1.22)	0.665	0.00	-	-	-	-
**Study methodology characteristics**	**Assay type**	Western blot	27	3,029	1.00	-		-	-	-	-
		ELISA	46	8,508	0.93 (0.82–1.05)	0.277		-	-	-	-
		Others	22	1,710	1.05 (0.90–1.22)	0.496	4.83	-	-	-	-
	**Sample size**^d^	<100	13	791	1.00	-		-	-	-	-
		≥100	82	12,454	0.93 (0.75–1.08)	0.364	0.26	-	-	-	-
	**Sampling method**	Non-probability-based	69	8,536	1.00	-		-	-	-	-
		Probability-based	26	4,701	0.93 (0.82–1.45)	0.210	1.41	-	-	-	-
	**Response rate**	≥80	22	5,155	1.00	-		-	-	-	-
		Otherwise^e^	73	8,091	0.91 (0.80–1.03)	0.164	0.93	-	-	-	-
**Temporal measures**	**Year of publication category**	<2000	28	2,935	1.00	-		-	-	-	-
		2000–2009	32	3,844	0.87 (0.76–0.91)	0.053		-	-	-	-
		2010–2018	35	6,468	0.86 (0.75–0.70)	0.023	8.67	-	-	-	-
	**Year of data collection**		95	13,335	0.99 (0.99–1.00)	0.047	6.86	-	-	-	-
	**Year of publication**		95	13,335	0.99 (0.99–0.99)	0.035	7.66	0.99 (0.99–1.00)	0.389	0.99 (0.99–0.99)	0.043

^a^ Variance explained by the final multivariable model 1 (adjusted *R*^*2*^) = 42.82%.

^b^ Variance explained by the final multivariable model 2 (adjusted *R*^*2*^) = 69.57%.

^c^ Other includes one measure of a low income country (Haiti) and the measures extracted from studies including different countries.

^d^ Sample size denotes the sample size of the study population found in the original publication.

^e^ Otherwise indicates either response rate was <80% or response rate not included in the report.

Abbreviations: *ARR* = Adjusted risk ratio, CI = Confidence interval, ELISA = Enzyme-linked immunosorbent type-specific assay, HIC = High-income country, HSV-1 = Herpes simplex virus type 1, *RR* = Risk ratio, UMIC = Upper-middle-income country.

Since age bracket and age group are variables that are not independent of each other, two multivariable models were analyzed, each using one of these variables. For a similar consideration, the year of publication was included in the multivariable analyses, instead of year of data collection, given its more complete data. As for the multivariable analyses including the year of publication category, instead of the *linear* year of publication term, the results can be found in S3 Table.

The first model included age bracket, sex, population type, and year of publication. It explained 42.82% of the seroprevalence variation. In adults, seroprevalence was 1.39-fold (95% CI: 1.24–1.57) higher than that in children.

The second model included age group, sex, population type, and year of publication. It explained 69.57% of the seroprevalence variation. Compared to those aged ≤10, seroprevalence was 1.36-fold (95% CI: 1.19–1.56) higher in those aged 10–20, 1.44-fold (95% CI: 1.25–1.65) higher in those aged 20–30, 1.70-fold (95% CI: 1.47–1.97) higher in those aged 30–40, and 1.81-fold (95% CI: 1.58–2.08) higher in those aged ≥40. There was evidence here for a statistically-significant declining seroprevalence over time by 0.99-fold (95% CI: 0.99–0.99) per year, in contrast to the first model analysis (Table 3) and the analyses including the year of publication as a category (S3 Table), where the evidence for the decline in sero-prevalence did not reach statistical significance.

### HSV-1 virus isolation in genital ulcer disease and in genital herpes

Tables 4 and 5 summarize the extracted proportions of HSV-1 virus isolation in GUD (n = 5) and in genital herpes (n = 9), as well as their pooled mean estimates.

**Table 4:** Studies reporting proportions of HSV-1 virus isolation in clinically-diagnosed GUD and in clinically-diagnosed genital herpes in Latin America and the Caribbean.

Author, year	Year(s) of data collection	Country	Study site	Study design	Sampling method	Population	HSV-1 biological assay	Sample size	Proportion of HSV-1 isolation (%)
**HSV-1 virus isolation in clinically-diagnosed GUD (n = 5)**									
Gomes Naveca, 2013 [62]	2008	Brazil	Outpatient clinic	CS	Conv	Patients with GUD	PCR	15	6.6
Gomes Naveca, 2013 [62]	2008	Brazil	Outpatient clinic	CS	Conv	Patients with primary GUD	PCR	324	4.0
Gomes Naveca, 2013 [62]	2008	Brazil	Outpatient clinic	CS	Conv	Patients with recurrent GUD	PCR	95	1.1
Noda, 2016 [63]	2012	Cuba	Outpatient clinic	CS	Conv	Men with GUD	PCR	113	0.0
Valdespino-Gomez, 1995 [64]	1990	Mexico	Community	CS	Conv	FSWs with genital ulcers	IFA	71	0.0
**HSV-1 virus isolation in clinically-diagnosed genital herpes (n = 9)**									
Balachandran, 1982 [27]	-	Puerto Ricco	Outpatient clinic	CS	Conv	STI clinic attendees	IFA	12	8.3
Belli, 1990 [65]	1982–83	Argentina	Outpatient clinic	CS	Conv	Patients with genital herpes	IFA	25	20.0
Do Nascimento, 1998 [30]	1995	Brazil	Outpatient clinic	CS	Conv	HIV patients with genital herpes	PCR	36	5.0
Hun,1987 [66]	-	Costa Rica	Outpatient clinic	CS	Conv	STI clinic attendees	Culture	12	25.0
Prabhakar, 1987 [67]	1982	Jamaica	Outpatient clinic	CS	Conv	STI clinic attendees	IFA	40	0.0
Schultz, 1994 [68]	1988	Chile	Outpatient clinic	CS	Conv	Pregnant women with genital herpes	DFA	20	10.0
Suarez, 1988 [69]	1985	Chile	Outpatient clinic	CS	Conv	Patients with primary genital herpes	IFA	14	28.5
Suarez, 1988 [69]	1985	Chile	Outpatient clinic	CS	Conv	Patients with recurrent genital herpes	IFA	61	9.8
Suarez, 1989 [70]	1984	Chile	Outpatient clinic	CS	Conv	Women with genital herpes	DFA	13	23.1

Abbreviations: Conv = Convenience, CS = Cross sectional, DFA = Direct fluorescent assay, FSWs = Female sex workers, GUD = Genital ulcer disease, HSV-1 = Herpes simplex virus type 1, IFA = Indirect immunofluorescence assay, PCR = Polymerase chain reaction, RS = Random Sampling, STI = Sexually transmitted infection.

**Table 5:** Pooled proportions of HSV-1 virus isolation in clinically-diagnosed GUD and in clinically-diagnosed genital herpes in Latin America and the Caribbean.

Population type	Outcome measures	Samples	Proportion of HSV-1 isolation (%)		Pooled proportion of HSV-1 isolation (%)	Heterogeneity measures		
	Total n	Total N	Range	Median	Mean (95% CI)	Q^a^ (p-value)	I^2^^b^ (%) (95% CI)	Prediction Interval^c^ (%)
Patients with clinically-diagnosed GUD	5	618	0.0–6.6	1.1	0.9 (0.0–3.6)	12.9 (p = 0.0116)	69.1 (20.7–88.0)	0.0–14.6
Patients with clinically-diagnosed genital herpes	9	233	0.0–28.5	10.0	10.9 (4.4–19.4)	21.1 (p = 0.0069)	62.1 (21.7–81.6)	0.0–40.4

^a^ Q: The Cochran’s Q statistic is a measure assessing the existence of heterogeneity in pooled outcome measures, here proportions of HSV-1 virus isolation.

^b^ I^2^: A measure assessing the magnitude of between-study variation that is due to true differences in proportions of HSV-1 virus isolation across studies rather than sampling variation.

^c^ Prediction interval: A measure quantifying the distribution 95% interval of true proportions of HSV-1 virus isolation around the estimated pooled mean.

Abbreviations: CI = Confidence interval, GUD = Genital ulcer disease, HSV-1 = Herpes simplex virus type 1.

In GUD cases, the virus isolation proportion ranged between 0.0–6.6%, with a median of 1.1% and a pooled mean of 0.9% (95% CI: 0.0–3.6%). In genital herpes cases, the proportion ranged between 0.0–28.5%, with a median of 10.0% and a pooled mean of 10.9% (95% CI: 4.4–19.4%). Both meta-analyses of proportions showed strong evidence of heterogeneity (Table 5). Forest plots can be found in S2 Fig.

### Quality assessment

A total of 31 reports were included in the systematic review, while an additional 12 reports were excluded due to potential issues in their diagnostic method (Fig 2).

Summary of the precision and ROB assessments are in S4 Table. High precision was found in the majority of studies (62.9%). High ROB in the sampling method domain was found in the vast majority of studies (94.3%). Low ROB in the response rate domain was found in 25.7% of studies, while the remaining studies had a high ROB (2.9%), or an unclear ROB (71.4%).

Since none of the study characteristics of sample size, sampling method, and response rate were found associated with HSV-1 seroprevalence (Table 3), it is not likely that precision nor ROB have affected the results of the present study.

## Discussion

The systematic review and meta-analytics reported here indicate that HSV-1 infection is widely prevalent in Latin America and the Caribbean, at a seroprevalence level that is higher than that of the global population at 67% [1]. Nearly 60% of children and 90% of adults are infected, a higher seroprevalence than that in Western Countries [31] and Asia [8], though lower than that in Africa [32] and the Middle East and North Africa (MENA) [33]. Seroprevalence increased steadily with age, but most HSV-1 acquisitions still occurred in childhood (Tables 2 and 3).

Age was by far the strongest predictor of infection, explaining alone >50% of the seroprevalence variation (Table 3). Meanwhile, sex, clinical condition, and country’s income did not affect HSV-1 seroprevalence (Table 3), in broad agreement with the results of similar studies for Africa [32], Asia [8], and MENA [33]. These findings affirm the notion that HSV-1 is a truly general population infection, with largely homogenous exposure risk in the population.

There was evidence for a declining seroprevalence over the last three decades, but the exact effect size of the decline and nature of the decline (linear or not) are not yet certain with currently available data (Tables 2 and 3 and S3 Table). While seroprevalence declines have been also observed in North America and Europe [31, 34–41], no evidence for such declines was found in Africa [32], Asia [8], and MENA [33]. The large gap in HSV-1 seroprevalence between children and adults (Tables 2 and 3), supports also the interpretation of recent declines in seroprevalence, with the currently older cohorts experiencing higher infection risk in earlier times. As observed in North America [31], improvements in hygiene and standard of living may have driven the seroprevalence declines.

With this evidence for a possible slow transition in HSV-1 epidemiology in Latin America and the Caribbean, there is a cause for concern for genital herpes, as increasingly a larger fraction of adolescents may initiate sexual activity with no antibodies to protect them against acquiring HSV-1 sexually, and thus at risk of genital herpes. Indeed, we found evidence supporting a role for HSV-1 as the etiological cause of genital herpes (Tables 4 and 5), though at rates much lower than those observed in Western countries [4, 5, 7, 9–11] and Asia [8].

This study has limitations. Data were available only for 14 mostly populous countries (Tables 1 and 4), with no data found for the remaining 32 smaller countries. Studies varied in methods and quality and used different diagnostic assays, with potentially different sensitivity and specificity profiles [16, 17]. However, no effect was found on seroprevalence for assay type, sample size, sampling method, and response rate (Table 3), indicating that the variability in study methods may not have impacted the results and findings of the present study.

## Conclusions

As in North America, Europe, and Asia [5, 7–11, 31, 35, 42], there is evidence for a possible transitioning HSV-1 epidemiology in Latin America and the Caribbean, though at a slower rate and with still limited contribution for HSV-1 in genital herpes and as a sexually transmitted infection. HSV-1 seroprevalence appears to be declining, with the younger cohorts experiencing lower infection risk than those experienced by the younger cohorts in earlier times. Yet, HSV-1 persists as a widely prevalent infection in this region, with 60% of children and 90% of adults being infected. These findings support the need for surveillance to monitor trends in seroprevalence and genital herpes etiology, and highlight the need for a vaccine to prevent infection and associated disease burden.

## Supporting information

S1 Table Preferred Reporting Items for Systematic Reviews and Meta-analyses (PRISMA) checklist. (DOCX)

S2 Table Data sources and search criteria for systematically reviewing HSV-1 epidemiology in Latin America and the Caribbean. (DOCX)

S3 Table Multivariable meta-regression models for HSV-1 seroprevalence in Latin America and the Caribbean including the categorical stratification by year of publication. (DOCX)

S4 Table Summary of the precision assessment and risk of bias (ROB) assessment for the studies reporting HSV-1 seroprevalence in Latin America and the Caribbean. (DOCX)

S1 Box List of the 46 countries included in our definition for the Latin America and the Caribbean region. (DOCX)

S2 Box List of variables extracted from the relevant reports meeting the inclusion criteria. (DOCX)

S1 Fig Forest plots presenting the outcomes of the pooled mean HSV-1 seroprevalence among children and adult populations in Latin America and the Caribbean. (DOCX)

S2 Fig Forest plots presenting the outcomes of the pooled mean proportions of HSV-1 virus isolation in clinically-diagnosed genital ulcer disease and in clinically-diagnosed genital herpes in Latin America and the Caribbean. (DOCX)
