# Supplementary material for: Herpes simplex virus type 1 epidemiology in Latin America and the Caribbean: Systematic review and meta-analytics
Source: PLoS One. 2019 Apr 22;14(4):e0215487. doi: 10.1371/journal.pone.0215487 (PMC6476500; doi:10.1371/journal.pone.0215487)
Supplement: S2 Table — (DOCX) [file pone.0215487.s002.docx]

**S2 Table.** Data sources and search criteria for systematically reviewing HSV-1 epidemiology in Latin America and the Caribbean.

| **PubMed (last searched September 12, 2018):** |
| --- |
| (“Simplexvirus”[MeSH] OR “Herpesviridae”[MeSH] OR “Herpes Simplex”[MeSH] OR “Herpesvirus 1, Human”[MeSH]) OR (HSV type-1[Text] OR HSV type 1[Text] OR HSV1[Text] OR HSV-1[Text] OR HSV 1[Text] OR Human herpes virus[Text] OR Herpes simplex virus type 1[Text] OR Herpes simplex virus type-1[Text] OR herpes simplex virus 1[Text] OR herpes simplex virus-1[Text] OR herpes simplex type 1[Text] OR herpes simplex type-1[Text] OR herpes simplex 1[Text] OR herpes simplex-1[Text] OR Herpesvirus type 1[Text] OR Herpesvirus type-1[Text] OR Herpesvirus 1[Text] OR Herpesvirus-1[Text] OR Herpes virus type 1[Text] OR Herpes virus type-1[Text] OR Herpes virus 1[Text] OR Herpes virus-1[Text] OR genital herpes[Text] OR Herpes Genitalis[Text] OR Stomatitis Herpetic[Text] OR Herpes Labialis[Text]) AND (“Latin America”[MeSH] OR “Central America"[MeSH] OR “South America”[Mesh] OR “Caribbean Region”[MeSH]  OR “Mexico”[MeSH]) OR (Anguilla*[Text] OR Aruba*[Text] OR Antigua and Barbuda[Text] OR Argentin*[Text] OR Bahamas*[Text] OR Barbados*[Text] OR Beliz*[Text] OR Bermuda*[Text]  OR Bolivia*[Text] OR Brazil*[text] OR “British Virgin Islands”[Text] OR Latin America[Text]  OR Latin American*[Text] OR Caribbean*[Text] OR Cayman Islands[Text] OR Chile*[Text] OR Colombia*[Text] OR Costa Rica*[Text] OR Cuba*[Text] OR Curacao*[Text] OR Central America[Text] OR Central American*[text] OR Dominica*[Text] OR Dominican republic[Text] OR Ecuador*[Text] OR El Salvador[Text] OR French Guiana[Text] OR Grenad*[Text] OR Guadeloup*[Text] OR Guatemal*[Text] OR Guyan*[Text]  OR Haiti*[Text] OR Honduras*[Text] OR Jamaic*[Text] OR Martiniqu*[Text] OR Montserrat*[Text] OR Mexic*[Text] OR Nicaragua*[Text] OR Panama*[Text] OR Paraguay*[Text] OR Peru*[Text] OR Puerto Rico[Text] OR Puerto Rica*[text] OR Saint Kitts and Nevis[Text] OR Saint Lucia[Text] OR Saint Vincent and the Grenadines[Text] OR Suriname*[Text] OR Saint Martin[Text] OR Sint Maarten[Text] OR South America[Text] OR South American*[Text] OR Trinidad and Tobago[Text] OR Turks and Caicos[Text]  OR Uruguay*[Text] OR United States Virgin Islands[Text] OR Venezuel*[Text]) |
| **Embase (last searched September 12, 2018):** |
| (exp Herpes simplex / or exp herpesviridae or (Herpes simplex or Herpes simplex virus or HSV type-1 or HSV type 1 or HSV1 or HSV-1 or HSV 1 or human herpes virus or Herpes simplex virus type 1 or Herpes simplex virus type-1 or herpes simplex virus 1 or herpes simplex virus-1 or herpes simplex type 1 or herpes simplex type-1 or herpes simplex 1 or herpes simplex-1 or Herpesvirus type 1 or Herpesvirus type-1 or Herpesvirus 1 or Herpesvirus-1 or Herpes virus type 1 or Herpes virus type-1 or Herpes virus 1 or Herpes virus-1 or genital herpes or Herpes Genitalis or herpes labialis or herpetic stomatitis).mp. AND (exp "Antigua and Barbuda"/ or exp Argentina/ or exp Aruba/ or exp Bahamas/ or exp Barbados/ or exp Belize/ or exp Bolivia/ or exp Brazil/ or exp "Virgin Islands (British)"/ or exp Cayman Islands/ or exp Chile/ or exp Colombia/ or exp Costa Rica/ or exp Cuba/ or exp Curacao/ or exp Dominica/ or exp Dominican Republic/ or exp Ecuador/ or exp El Salvador/ or exp French Guiana/ or exp Grenada/ or exp Guadeloupe/ or exp Guatemala/ or exp Guyana/ or exp Haiti/ or exp Honduras/ or exp Jamaica/ or exp Martinique/ or exp Mexico/ or exp Montserrat/ or exp Nicaragua/ or exp Panama/ or exp Paraguay/ or exp Peru/ or exp Puerto Rico/ or exp Saint Lucia/ or exp "saint martin (dutch)"/ or exp "saint martin (french)"/ or exp Suriname/) or (exp "Trinidad and Tobago"/ or exp "Virgin Islands (U.S.)"/ or exp Uruguay/ or exp Venezuela/ or exp South America/ or exp Central America/ or exp Caribbean/ or exp "Caribbean (person)"/ or exp Caribbean Netherlands/ or exp Caribbean Islands/ or exp South American/ or exp Central American/ or exp Latin America/) or (Antigua or Argentina or Argentinian or Aruba or Aruban or Bahamas or Belize or belizian or Bolivia or Bolivian or Brazil or Brazilian or British virgin islands or Cayman islands or Chile or Chilean or Colombia or Colombian or Costa Rica or costa Rican or Cuba or Cuban or Curacao or Dominica or Dominican or Dominican republic or Ecuador or Ecuadorian or el Salvador or el Salvadorian ).mp. or (French Guiana or Grenada or Guadeloupe or Guatemala or Guatemalan or Guyana or Haitian or Honduras or Honduran or Jamaica or Jamaican or Martinique or Mexico or Mexican or Montserrat or Nicaragua or Nicaraguan or panama or Panamanian or Paraguay or Paraguayans or Peru or Peruvian or Puerto Rico or Puerto Ricans or saint Lucia or saint Lucian or Latin American or south American or central american).mp. or ((Turks and caicos) or (saint vincents and the grenadines) or (saint kitts and the nevis)).mp. |
| **LILACS (last searched September 12, 2018):** |
| tw:((tw:(herpes)) OR (tw:(herpesvirus 1)) OR (tw:(herpes simplex)) OR (tw:(hsv type-1)) OR (tw:(hsv type 1)) OR (tw:(hsv1)) OR (tw:(hsv-1)) OR (tw:(hsv 1)) OR (tw:(human herpes virus)) OR (tw:(herpes simplex virus type 1)) OR (tw:(herpes simplex virus type-1)) OR (tw:(herpes simplex virus 1)) OR (tw:(herpes simplex virus-1)) OR (tw:(herpes simplex type 1)) OR (tw:(herpes simplex type-1)) OR (tw:(herpes simplex 1)) OR (tw:(herpes simplex-1)) OR (tw:(herpesvirus type 1)) OR (tw:(herpesvirus type-1)) OR (tw:(herpesvirus 1)) OR (tw:(herpesvirus-1)) OR (tw:(herpes virus type 1)) OR (tw:(herpes virus type-1)) OR (tw:(herpes virus 1)) OR (tw:(herpes virus-1)) OR (tw:(genital herpes)) OR (tw:(herpes genitalis)) OR (tw:(stomatitis herpetic))) AND (instance:"regional") AND ( pais_assunto:("america do sul" OR "brasil" OR "oceania" OR "mexico" OR "argentina" OR "caribe ingles" OR "caribe" OR "chile" OR "america central" OR "colombia" OR "venezuela" OR "jamaica" OR "peru" OR "cuba" OR "costa rica" OR "puerto rico" OR "panama" OR "bolivia" OR "haiti" OR "ecuador" OR "guyana francesa" OR "guyana" OR "barbados" OR "trinidad y tobago" OR "uruguay" OR "honduras" OR "el salvador" OR "guatemala" OR "paraguay" OR "nicaragua" OR "republica dominicana" OR "dominica" OR "Bahamas" OR "grenada" OR "martinica" OR "santa lucia" OR "suriname")) |

Abbreviations: HSV-1 = Herpes simplex virus type 1
