# Supplementary material for: Herpes simplex virus type 1 epidemiology in Latin America and the Caribbean: Systematic review and meta-analytics
Source: PLoS One. 2019 Apr 22;14(4):e0215487. doi: 10.1371/journal.pone.0215487 (PMC6476500; doi:10.1371/journal.pone.0215487)
Supplement: S3 Table — (DOCX) [file pone.0215487.s003.docx]

**S3 Table.** Multivariable meta-regression models for HSV-1 seroprevalence in Latin America and the Caribbean including the categorical stratification by year of publication.

|  | | | **Outcome measures** | **Samples** | **Univariable analysis** | | | **Multivariable analysis^a^** | | | |
| --- | --- | --- | --- | --- | --- | --- | --- | --- | --- | --- | --- |
|  |  |  |  |  |  |  |  | **Model 1^a^** | | **Model 2^b^** | |
|  | | | **Total n** | **Total N** | ***RR* (95%CI)** | **p-value** | **Adjusted R^2^ (%)** | ***ARR* (95%CI)** | **p-value** | ***ARR* (95%CI)** | **p-value** |
| **Population Characteristics** | **Age bracket** | Children | 19 | 2,026 | 1.00 | - |  | 1.00 | - | - | - |
|  |  | Adults | 73 | 10,690 | 1.45 (1.29-1.64) | <0.001 |  | 1.39 (1.24-1.57) | <0.001 | - | - |
|  |  | Age-mixed | 3 | 531 | 1.35 (1.04-1.75) | 0.022 | 35.37 | 1.30 (1.00-1.68) | 0.043 | - | - |
|  | **Age group** | ≤10 | 14 | 1,438 | 1.00 | - |  | - | - | 1.00 | - |
|  |  | 10-20 | 17 | 2,294 | 1.44 (1.24-1.67) | <0.001 |  | - | - | 1.37 (1.20-1.57) | <0.001 |
|  |  | 20-30 | 12 | 1,926 | 1.53 (1.31-1.79) | <0.001 |  | - | - | 1.44 (1.25-1.66) | <0.001 |
|  |  | 30-40 | 9 | 1,181 | 1.76 (1.49-2.08) | <0.001 |  | - | - | 1.70 (1.46-1.97) | <0.001 |
|  |  | ≥40 | 11 | 2,128 | 1.81 (1.54-2.11) | <0.001 |  | - | - | 1.81 (1.57-2.08) | <0.001 |
|  |  | Mixed | 32 | 4,280 | 1.68 (1.47-1.93) | <0.001 | 53.99 | - | - | 1.53 (1.34-1.74) | <0.001 |
|  | **Sex** | Female | 46 | 6,723 | 1.00 | - |  | 1.00 | - | 1.00 | - |
|  |  | Male | 17 | 2,771 | 0.86 (0.75-1.00) | 0.053 |  | 0.96 (0.84-1.09) | 0.528 | 0.96 (0.86-1.07) | 0.488 |
|  |  | Mixed | 32 | 3,751 | 0.93 (0.82-1.05) | 0.277 | 3.62 | 1.03 (0.93-1.14) | 0.524 | 1.01 (0.93-1.10) | 0.718 |
|  | **Population type** | Healthy | 72 | 10,456 | 1.00 | - |  | 1.00 | - | 1.00 | - |
|  |  | Clinical | 7 | 530 | 1.19 (0.99-1.43) | 0.062 |  | 1.10 (0.93-1.29) | 0.260 | 1.12 (0.97-1.29) | 0.118 |
|  |  | Other | 16 | 2,258 | 1.28 (1.12-1.45) | <0.001 | 17.13 | 1.15 (1.00-1.33) | 0.041 | 1.18 (1.05-1.32) | 0.005 |
|  | **Country’s income** | UMIC | 85 | 11,891 | 1.00 | - |  | - | - | - | - |
|  |  | HIC | 5 | 925 | 1.12 (0.88-1.42) | 0.324 |  | - | - | - | - |
|  |  | Other^c^ | 5 | 429 | 0.95 (0.73-1.22) | 0.665 | 0.00 | - | - | - | - |
| **Study methodology characteristics** | **Assay type** | Western blot | 27 | 3,029 | 1.00 | - |  | - | - | - | - |
|  |  | ELISA | 46 | 8,508 | 0.93 (0.82-1.05) | 0.277 |  | - | - | - | - |
|  |  | Others | 22 | 1,710 | 1.05 (0.90-1.22) | 0.496 | 4.83 | - | - | - | - |
|  | **Sample size^d^** | <100 | 13 | 791 | 1.00 | - |  | - | - | - | - |
|  |  | ≥100 | 82 | 12,454 | 0.93 (0.75-1.08) | 0.364 | 0.26 | - | - | - | - |
|  | **Sampling method** | Non-probability-based | 69 | 8,536 | 1.00 | - |  | - | - | - | - |
|  |  | Probability-based | 26 | 4,701 | 0.93 (0.82-1.45) | 0.210 | 1.41 | - | - | - | - |
|  | **Response rate** | ≥80 | 22 | 5,155 | 1.00 | - |  | - | - | - | - |
|  |  | Otherwise^e^ | 73 | 8,091 | 0.91 (0.80-1.03) | 0.164 | 0.93 | - | - | - | - |
| **Temporal measures** | **Year of publication category** | <2000 | 28 | 2,935 | 1.00 | - |  | 1.00 | - | 1.00 | - |
|  |  | 2000-2009 | 32 | 3,844 | 0.87 (0.76-0.91) | 0.053 |  | 0.96 (0.85-1.09) | 0.575 | 0.95 (0.85-1.05) | 0.331 |
|  |  | 2010-2018 | 35 | 6,468 | 0.86 (0.75-0.70) | 0.023 | 8.67 | 0.96 (0.84-1.10) | 0.617 | 0.93 (0.83-1.04) | 0.228 |
|  | **Year of data collection** | | 95 | 13,335 | 0.99 (0.99-1.00) | 0.047 | 6.86 | - | - | - | - |
|  | **Year of publication** | | 95 | 13,335 | 0.99 (0.99-0.99) | 0.035 | 7.66 | - | - | - | - |

^a^ Variance explained by the final multivariable model 1 (adjusted *R^2^*) = 41.17%.

^b^ Variance explained by the final multivariable model 2 (adjusted *R^2^*) = 66.33%.

^c^ Other includes one measure of a low income country (Haiti) and the measures extracted from studies including different countries.

^d^ Sample size denotes the sample size of the study population found in the original publication.

^e^ Otherwise indicates either response rate was <80% or response rate not included in the report.
