# Supplementary material for: Herpes simplex virus type 1 epidemiology in Latin America and the Caribbean: Systematic review and meta-analytics
Source: PLoS One. 2019 Apr 22;14(4):e0215487. doi: 10.1371/journal.pone.0215487 (PMC6476500; doi:10.1371/journal.pone.0215487)
Supplement: S4 Table — (DOCX) [file pone.0215487.s004.docx]

**S4 Table.** Summary of the precision assessment and risk of bias (ROB) assessment for the studies reporting HSV-1 seroprevalence in Latin America and the Caribbean.

| **Quality assessment** | **HSV-1 seroprevalence measures** | |
| --- | --- | --- |
|  | **Number of studies** | **%** |
| **Precision of seroprevalence measures^a^** | | |
| Low precision | 13 | 37.1 |
| High precision | 22 | 62.9 |
| **Risk of bias quality domain^b^** | | |
| **Sampling method** | | |
| Low risk of bias | 2 | 5.71 |
| High risk of bias | 33 | 94.3 |
| **Response rate** | | |
| Low risk of bias | 9 | 25.7 |
| High risk of bias | 1 | 2.9 |
| Unclear risk of bias | 25 | 71.4 |
| **Summary of the risk of bias assessment** | | |
| **Low risk of bias** |  |  |
| In at least one quality domain | 10 | 28.6 |
| In both quality domains | 1 | 2.8 |
| **High risk of bias** |  |  |
| In at least one quality domain | 34 | 97.1 |
| In both quality domains | 0 | 0.0 |
| **Seroprevalence studies where risk of bias assessment was possible** | **35** | **100** |

^a^ Precision was assessed based on the overall sample size (not each stratum subsample size) of the study as reported in the record/publication.

^b^ Risk of bias was assessed based on the overall sample size (not each stratum subsample size) of the study as reported in the record/publication.

Abbreviations: HSV-1 = Herpes simplex virus type 1.
