# Supplementary material for: Herpes simplex virus type 1 epidemiology in Latin America and the Caribbean: Systematic review and meta-analytics
Source: PLoS One. 2019 Apr 22;14(4):e0215487. doi: 10.1371/journal.pone.0215487 (PMC6476500; doi:10.1371/journal.pone.0215487)
Supplement: S1 Box — (DOCX) [file pone.0215487.s005.docx]

**S1 Box.** List of the 46 countries included in our definition for the Latin America and the Caribbean region.
