# Supplementary material for: Herpes simplex virus type 1 epidemiology in Latin America and the Caribbean: Systematic review and meta-analytics
Source: PLoS One. 2019 Apr 22;14(4):e0215487. doi: 10.1371/journal.pone.0215487 (PMC6476500; doi:10.1371/journal.pone.0215487)
Supplement: S2 Box — (DOCX) [file pone.0215487.s006.docx]

**S2 Box.** List of variables extracted from the relevant reports meeting the inclusion criteria.


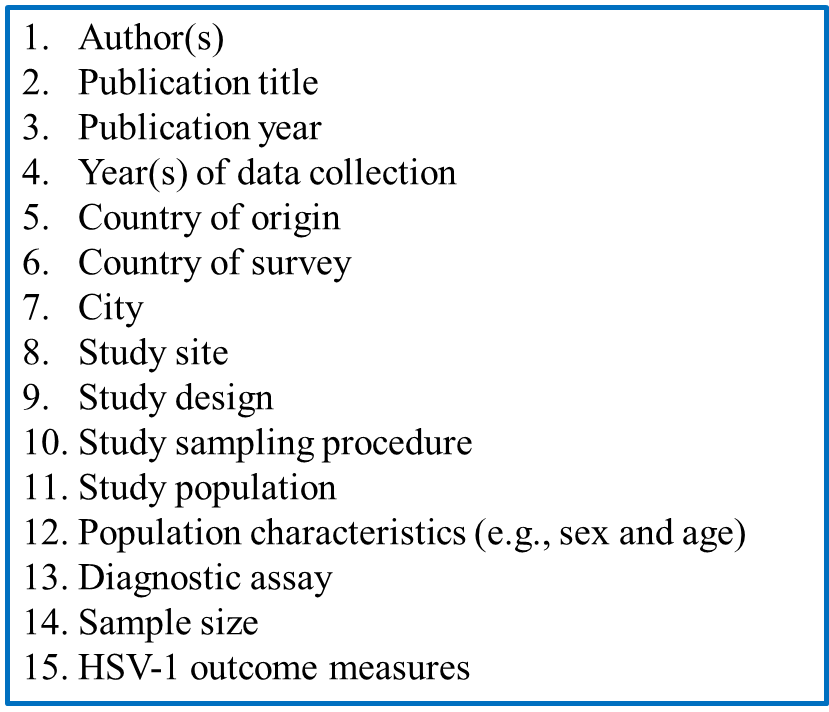


Abbreviations: HSV-1 = Herpes simplex virus type 1
