# Supplementary material for: Herpes simplex virus type 1 epidemiology in Latin America and the Caribbean: Systematic review and meta-analytics
Source: PLoS One. 2019 Apr 22;14(4):e0215487. doi: 10.1371/journal.pone.0215487 (PMC6476500; doi:10.1371/journal.pone.0215487)
Supplement: S1 Fig — (DOCX) [file pone.0215487.s007.docx]

**S1 Fig.** Forest plots presenting the outcomes of the pooled mean HSV-1 seroprevalence among children and adult populations in Latin America and the Caribbean.

1. All children populations


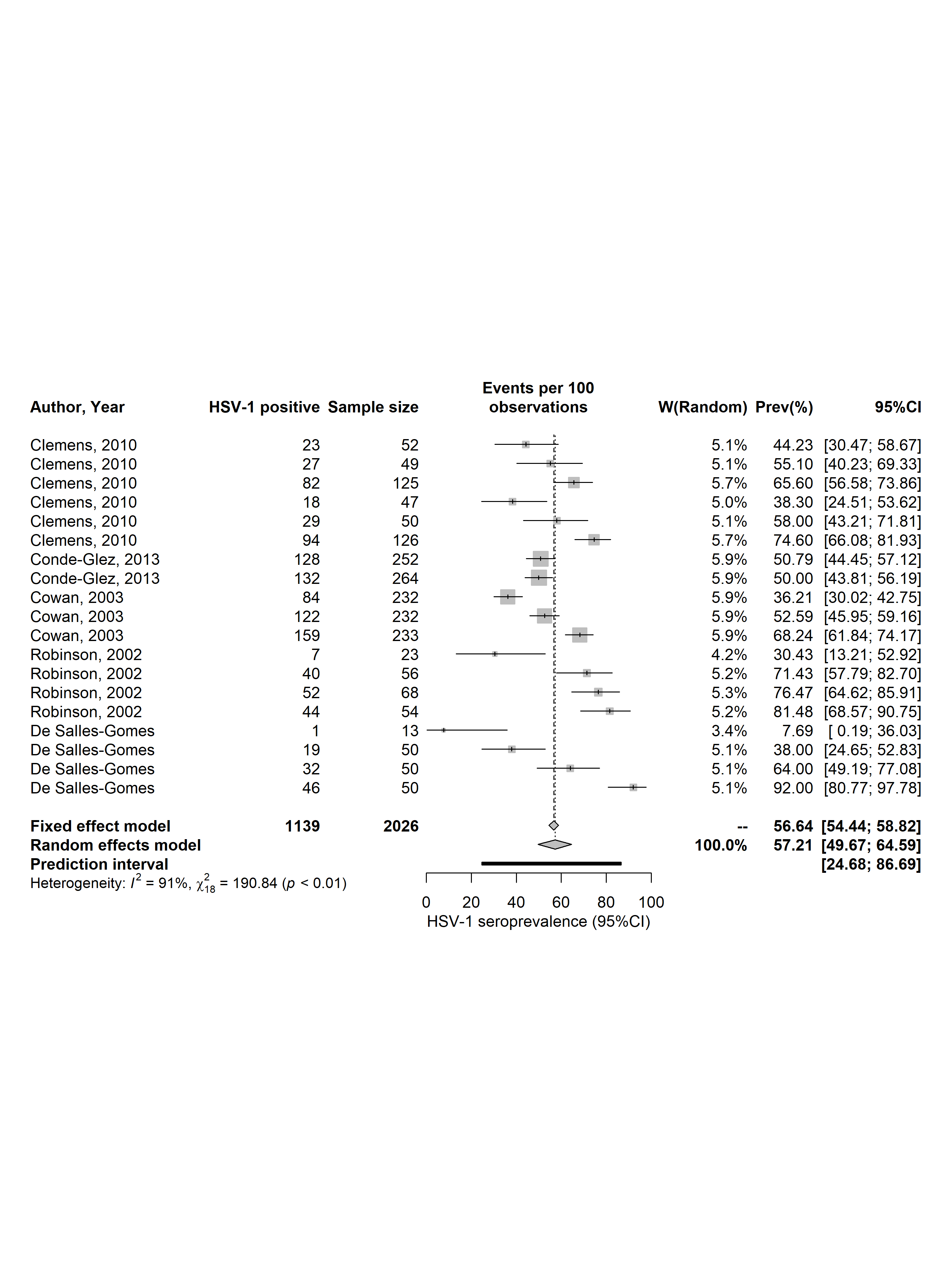

Abbreviations: HSV-1 = Herpes simplex virus type 1.

1. All adult populations


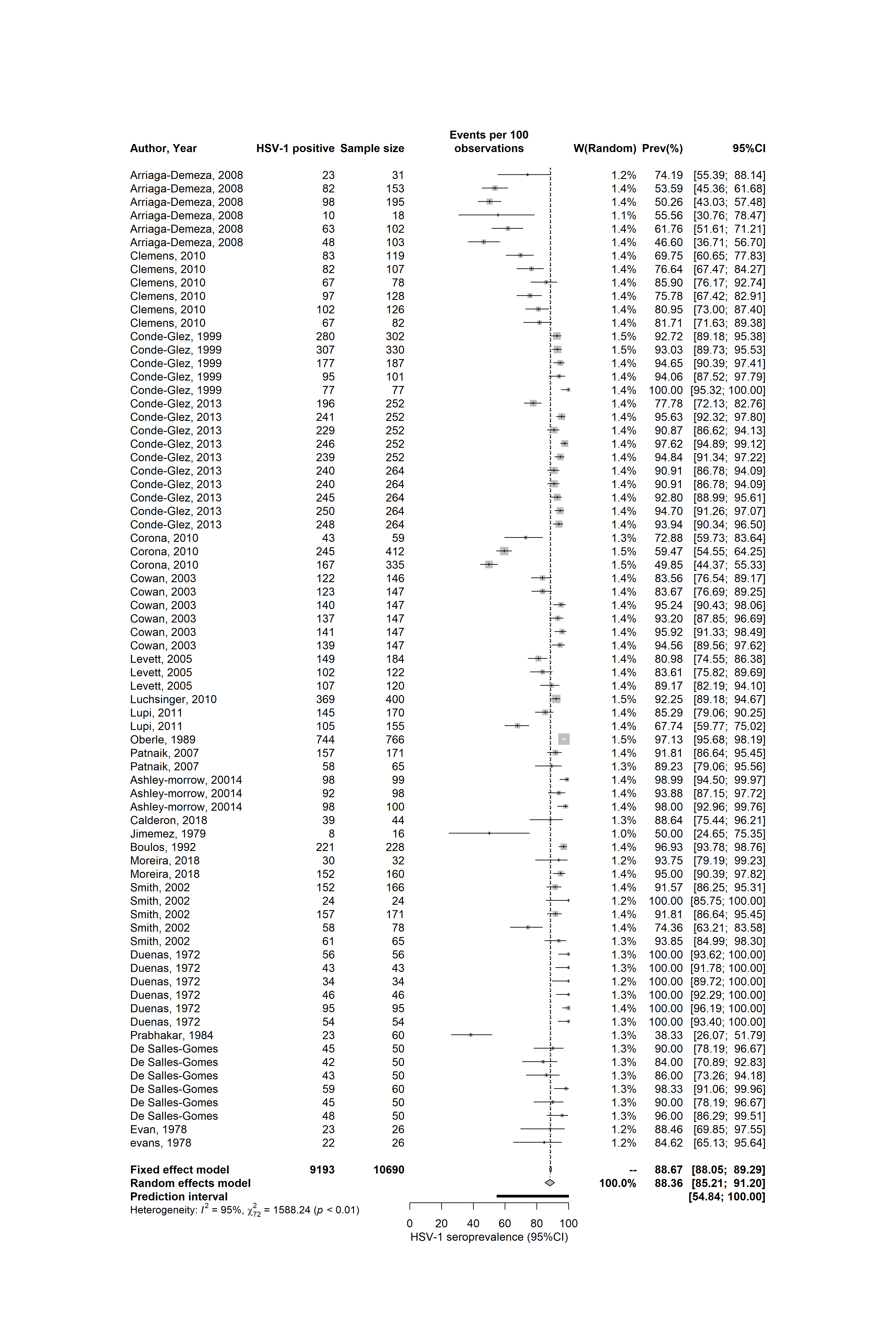


Abbreviations: HSV-1 = Herpes simplex virus type 1.
